# A 2‐Tyr‐1‐carboxylate Mononuclear Iron Center Forms the Active Site of a *Paracoccus* Dimethylformamidase

**DOI:** 10.1002/anie.202005332

**Published:** 2020-06-30

**Authors:** Chetan Kumar Arya, Swati Yadav, Jonathan Fine, Ana Casanal, Gaurav Chopra, Gurunath Ramanathan, Kutti R. Vinothkumar, Ramaswamy Subramanian

**Affiliations:** ^1^ Department of Chemistry Indian Institute of Technology Kanpur India; ^2^ Institute for Stem Cell Science and Regenerative Medicine Bangalore India; ^3^ National Center for Biological Sciences-TIFR GKVK Post Bangalore India; ^4^ Department of Chemistry Purdue University West Lafayette IN USA; ^5^ MRC Laboratory of Molecular Biology Cambridge UK; ^6^ Department of Biological Sciences and Weldon School of Biomedical Engineering Purdue University West Lafayette IN USA

**Keywords:** amide bond hydrolysis, bioremediation, dimethylforamamidase, metalloenzymes, protein structure

## Abstract

N,N‐dimethyl formamide (DMF) is an extensively used organic solvent but is also a potent pollutant. Certain bacterial species from genera such as Paracoccus, Pseudomonas, and Alcaligenes have evolved to use DMF as a sole carbon and nitrogen source for growth via degradation by a dimethylformamidase (DMFase). We show that DMFase from *Paracoccus* sp. strain DMF is a halophilic and thermostable enzyme comprising a multimeric complex of the α_2_β_2_ or (α_2_β_2_)_2_ type. One of the three domains of the large subunit and the small subunit are hitherto undescribed protein folds of unknown evolutionary origin. The active site consists of a mononuclear iron coordinated by two Tyr side‐chain phenolates and one carboxylate from Glu. The Fe^3+^ ion in the active site catalyzes the hydrolytic cleavage of the amide bond in DMF. Kinetic characterization reveals that the enzyme shows cooperativity between subunits, and mutagenesis and structural data provide clues to the catalytic mechanism.

## Graphical abstract

**Break it down again**: Certain bacteria can break down *N*,*N*‐dimethyl formamide (DMF) through the action of a dimethylformamidase (DMFase). The structure and mechanism of a DMFase from a bacterium of the genus *Paracoccus* are presented. The enzyme comprises a multimeric α_2_β_2_ or (α_2_β_2_)_2_ complex, and the active site consists of Fe^III^ coordinated by two Tyr side‐chain phenolates and one carboxylate from Glu in an unusual square pyramidal geometry.

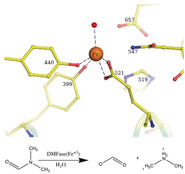

## Introduction

Dimethylformamide (DMF) is an organic solvent that is commonly used in the chemical, leather, printing, and petrochemical industries.^[1]^ The extent of its usage is evident from the quantity of its production.[^2^, ^3^] Its polar nature accords properties similar to water, and its physicochemical features (e.g., miscibility in many organic solvents and water, a high boiling point of 153 °C) make it very versatile.[^1^, ^4^, ^5^, ^6^] It is a known hepatotoxic and ecotoxic agent.[^7^, ^8^] DMF is remarkably resilient to chemical and photochemical decomposition, thus making it a potent pollutant. Biodegradation has emerged as a strategy for its removal from the environment.^[9]^ Despite the short history of DMF on earth (it was introduced in 1893), several microbes that can grow using DMF as the sole carbon source have been described, with most of them belonging to the phylum Proteobacteria (*Pseudomonas*, *Alcaligenes, Orchobactrum* sp., and *Paracoccus* species).[^10^, ^11^, ^12^, ^13^, ^14^] *Pseudomonas* species use an oxidative demethylation pathway to convert DMF into formamide. The product is subsequently converted into ammonia and formate by the enzyme formamidase.[^15^, ^16^] Other organisms encode an *N*,*N*‐dimethylformamidase (DMFase) that catalyzes the formation of formate and dimethylamine.^[16]^ The DMFases from a few different organisms that have been characterized comprise two polypeptide chains: a smaller polypeptide of 15 kDa, and a larger polypeptide of 85 kDa.[^3^, ^15^, ^16^] Sequence‐based homology searches do not show any similarity to other ubiquitous amidohydrolase. The nature of the active site and the mechanism has remained a puzzle. Herein, we describe the structure of a DMFase from *Paracoccus sp*. strain DMF(parDMFase) determined by electron cryomicroscopy (cryoEM). The structure is supported by functional studies to provide a molecular understanding of how the enzyme has evolved to create an active‐site environment to break down a highly stable amide bond under harsh conditions of elevated temperatures, high salt concentrations, and an aprotic solvent.

## Results and Discussion

### Biochemical Characterization

**Figure 1:**
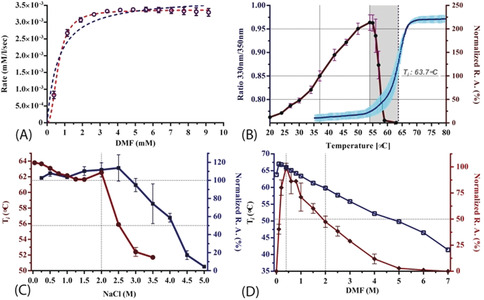
A) Real‐time ITC kinetic assay of the parDMFase. All data were fitted using GraphPad Prism v6.0. The blue data lines are fit to classical Michaelis–Menten equation (*V*_max_=0.0038±8.1E‐05; *K*_m_=0.63±0.08; R^2^=0.88) and red lines are fit to a model with a Hill co‐efficient (*V*_max_=0.0034±2.2E‐05; H≈2.0; *K*_half_=0.632±0.012; *K*_prime_=0.40; R^2^=0.98). The data suggest intramolecular cooperativity between two active sites within the dimer with a Hill's coefficient of 2 (*H*=2). B) Hydrolytic activity of the parDMFase at different temperatures. Relative catalytic activity (R.A, red line) was measured with respect to amidase activity at 310 K. The raw thermal unfolding of parDMFase (blue line) is shown by intrinsic fluorescence response with increased temperature. C) Hydrolytic activity of the parDMFase at different NaCl concentrations. The *T*_i_ of the enzymes incubated in different salt concentrations is plotted (red line) with respective relative amidase activities (blue line). D) Effect of DMF as an organic solvent on the catalytic activity and stability of the parDMFase. The T_*i*_ of the enzymes incubated in different DMF concentrations are plotted (blue line) with respective relative amidase activities (red line).

We purified the DMFase enzyme from a native *Paracoccus* species strain DMF and also expressed the gene recombinantly in *E.coli*. The kinetic parameters of the native and recombinant enzyme samples were similar for the substrate DMF (Figures S1, S2 and Table S1 in the Supporting Information). The data from the experiments are a better fit to the Hill equation (with a Hill coefficient of 2) rather than to a classical Michaelis–Menten equation, thus suggesting cooperativity between the subunits (Figure 1 A). The steady‐state kinetics data also show that the native and recombinant enzymes have similar *V*_max_ and *K*_m_ values to the DMFases from other organisms.[^3^, ^14^, ^17^] A thermal shift assay gave an optimum *T*_m_ of 64 °C in 250 mm NaCl for parDMFase, the revealing that it is a thermotolerant enzyme. The peak activity of the *Paracoccus*‐sourced enzyme is at 54 °C (Figure 1 B). The temperature dependence of the kinetic and thermodynamic parameters show reduced enzymatic activity at low temperatures (≤20 °C). We then tested the halo‐tolerant nature of the parDMFase by measuring the enzyme activity^[18]^ at increasing salt concentrations. Optimal hydrolase activity for the parDMFase was detected at 2.5 m NaCl (Figure 1 C). At this salt concentration, the temperature of inversion (*T*_i_) is 56 °C. When the activity and stability of parDMFase were checked at different concentrations of DMF, the enzyme was most active at 0.4 m DMF (Figure 1 D). The enzyme seems to have evolved to work best at 56 °C, 2.0 m salt, and 0. 4 m DMF in vitro; possibly similar conditions in which the bacterium survives in the effluent waste‐water sludge near tanneries at Kanpur. The substrate specificity of the parDMFase against compounds such as formamide, acetamide, *N*‐methylformamide (NMF), *N*‐ethylformamide (NEF), dimethylacetamide (DMAc), benzamide, n‐hexanamide, and urea was studied using real‐time ^1^H NMR based kinetic assays. As expected, the enzyme exhibits the highest *V*_max_ for DMF. Formamide, NEF, and NMF were also hydrolyzed with the high rate observed for NMF. However, other substrates yielded no detectable products, thus indicating that substitutions at the carbon are not preferred (Table 1).

**Table 1:** Kinetic properties of the parDMFase with different substrates. The following substrates showed no activity: benzamide, acrylamide, *n*‐hexanamide, urea, and acetamide.

Substrate	Structure	*V*_max_	*K*_m_	*k*_cat_	Catalytic efficiency
DMF	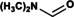	4.36	0.85	218	256.5
Formamide	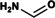	0.52	0.13	0.5	4.05
NMF	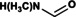	1.85	0.03	185	6166.7
NEF	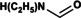	1.07	0.05	107	2104
DMAc	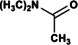	0.3	*N.A*.	*N.A*.	*N.A*.

### Structure Determination by Electron Cryomicroscopy

**Figure 2:**
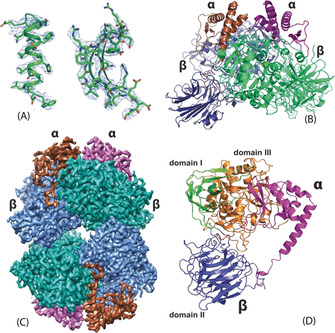
A) Representative regions of the electron density map at approximately 3 Å resolution that allowed tracing of the entire chain. B) Cartoon representation of the dimeric form. The large subunits are shown in green and blue and the small subunits in brown and purple. C) CryoEM map of the tetrameric form of the parDMFase made of two dimers of dimers. The large subunits are colored in blue and cyan and the small subunits in brown and purple. D) The structure of the unique polypeptide units that form the smallest structural unit, the αβ heterodimer. The small subunit is in purple, and the three domains of the large subunit are in green, orange‐brown, and blue.

**Figure 3:**
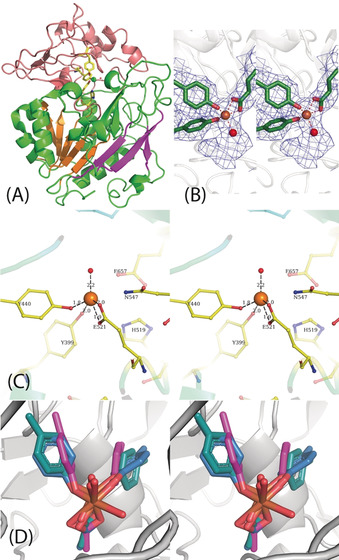
A) Domain III is shown with the active site. The five parallel β‐strands are in orange. The active site residues are shown as stick representations, and the Fe and liganded water molecules are shown as spheres. B) Stereoview of the cryo‐EM map around the active site with the modeled Fe, ligands, and a single water molecule. C) Stereoview of the residues in the active site including the essential His 519 and N547. D) Stereoview of the Fe site modeled from the purple orientations (found from the structures determined) and the relaxed orientations of the ligands after minimization by DFT. The significant rearrangement of the tyrosine suggests the strain in the active site.

When the purified native or recombinant parDMFase was imaged on ice by electron cryo‐microscopy (cryoEM), the micrographs unambiguously revealed two populations: a smaller molecule measuring approximately 100 Å diameter and a larger structure of approximately 150 Å in diameter could be identified. Subsequent 2D classification showed that the smaller unit was a dimer, and the larger structure was a dimer of dimers. The dimer of the parDMFase is made of two α and two β subunits with a total mass of 200 kDa. The dimer of dimers (called tetramer here) has a total mass of 400 kDa. The initial data we collected had a mixture of populations (Figure S3 A, B in the Supporting Information), and the reconstructions resulted in a 3.4 Å map for the dimer and a 3.8 Å map for the tetramer. We noted that the salt concentration in the buffer shifts the oligomer (dimer/tetramer) equilibrium. In the presence of low salt, the enzyme is predominantly in the dimeric form (Figure S3 C, D), and this equilibrium shifted to the tetramer at higher salt concentrations (≥200 mm; Figure 3 E, F). Subsequently, we collected two data sets with no salt and 200 mm NaCl yielding reconstructions with an overall resolution of 3 Å (dimer) and 2.8 Å (tetramer), respectively (Figures S4, 5 and Table S2). The local resolution plot of both maps showed that much of the reconstruction was resolved between 2.5–3.5 Å. The high quality of the maps allowed de novo tracing of polypeptide chains of both the large and small subunits (Figure 2 A). We used the dimer map for the initial tracing, and the final model is shown in Figure 2 B. The model from the dimer was used to obtain the tetramer model (Figure 2 C). Independently, we also determined the crystal structure of the parDMFase by using a model derived from cryoEM for molecular replacement (For details, see Methods section in Supporting Information). The overall structure from both these techniques is similar.

### The Structure of the parDMFase Reveals Two New Folds

The smallest unit is an αβ dimer (Figure 2 D). The large subunit consists of three different domains (Figure 2 D). Using PDBeFOLD,^[19]^ we identified three domains. Domain I comprises residues 1–73 and 338–383, and it adopts an immunoglobulin (IgG)‐like fold. Domain II comprises residues 74–337 and adopts the pentraxin fold. Domain III comprises residues 384–761 and is a hitherto undescribed fold (Figure 3 A). The core of the domain III fold itself can be described as α/β/α fold, with five β‐strands that are parallel and sandwiched between α‐helices. Such an arrangement is seen in the ThuA‐like family of proteins. These arrangements are classified as part of the larger family of glutamine amidotransferases.^[20]^ The five conserved β‐strands plus the three extra β‐strands together form a sheet that forms the inside of the sandwich (Figure 3 B). The connecting region between the β‐strands forms a sub‐domain made up of four anti‐parallel β‐strands that cap the structure. Domains I and III show a significant number of interactions, but the interaction of domain II with other domains of the monomer is somewhat limited (Figure 2 D).

The small subunit of the parDMFase consists of four α‐helices and two β‐strands. There are two long helices at the N‐ and C‐termini, with the N‐terminal helix wedging into the interface between the two large subunits (Figure 2 B & C). As a whole, the small subunit is also an undescribed fold. Most of the interactions of the small subunit are with domain III of the large subunit (Figure 2 D). The N‐terminal residues of the small subunit interact with domain II and seem to stabilize the tertiary structure by preventing this domain from adopting other orientations with respect to domains I and III. The large subunit, when expressed and purified without the small unit, was not enzymatically active. The size exclusion chromatography (SEC) profile suggests that it most likely exists as a dimer, albeit with a lower *T*_m_ (46 °C) than the α_2_β_2_ enzyme (data not shown). Thus, the small subunit most likely plays a role in structural stabilization.

### A Distinctive Metal Site

The initial EM map clearly showed a large density around residues Y440, Y399, and E521 that could not be accounted for by amino acids (Figure 3 B). We predicted this to be the active site, which is buried in domain III. The exact nature of the metal ion as Fe was confirmed by X‐ray fluorescence spectroscopy at the synchrotron (Supporting Information Figure 6 A). EPR spectra collected on the wild‐type protein suggested that under the conditions of purification and storage of the protein, the iron is mostly in the Fe^3+^ high‐spin state (Figure S6 B). The mononuclear Fe atom is tetra‐coordinated with the protein. It is coordinated to two phenolates of tyrosine residues (Y399 and Y440 serve as monodentate ligands), and a glutamic acid carboxylate side chain (E521 serves as a bidentate ligand; Figure 3 C). A histidine residue (H519) is observed close to the active site (Figure 3 C). However, in the model derived, this histidine residue does not coordinate with the Fe^3+^ ion in the active site. The distance between the N*ϵ*2 atom of H519 and Fe^3+^ is approximately 3.5 Å. The mutants Y440A and E521A were catalytically inactive. Structures of these mutant enzymes determined by cryoEM show a loss of Fe atoms (Figure S7 and Table S2). We also mutated residues H519 and S395, which are close to the metal‐binding center. Of these, S395A showed comparable activity to the wild‐type parDMFase, but the H519A mutant was catalytically inactive. Despite not coordinating Fe^3+^, it appears that H519 plays a major role in catalysis.

The coordination of Fe^3+^ is strained and is close to a square pyramidal geometry with two vacant sites (in the absence of modeled water). There is density at the active site that can accommodate at least one water molecule, which was modeled (Figure 3 A).

There are around 46 entries in the Protein Data Bank (PDB) in which tyrosine residues are coordinating ligands to a mononuclear Fe atom. The architecture of the two tyrosines binding to the Fe^3+^ ion is similar to that present in the structure of the ferric binding proteins from *Nisseria gonorrhoeae* (PDB ID: 1d9y) and *Yersinia enterocolitica* (PDB ID: 1xvx).^[21]^ Both of these also have a histidine coordinating the ligand and are not enzymes. The architecture with two tyrosine phenolates and a glutamic acid side‐chain carboxylate, as observed here, is unique.

To confirm that the chemical environment around the iron atom is indeed strained, we calculated the electronic energy of the iron atom and its surrounding environment using the experimentally observed structure (Figure 3 B) as the starting model. We compared this strain energy to the energy of a relaxed system (Figure 3 D). Electronic energies were calculated using density functional theory (DFT) with the B3LYP functional[^20^, ^21^, ^22^] and the 6–311G(d) basis set in Gaussian16^23^ (Figure S8). The difference in the energies obtained between the strained and relaxed octahedral states with Fe^3+^ ions is −222.01 kJ mol^−1^ (Table S3 in the Supporting information). The difference in energy between DMF and the first transition state of decomposition is 156.0 kJ mol^−1^. The difference in energy for the first intermediate is 27.4 kJ mol^−1^ (see Table S4 and Figure S9), thus suggesting that relief of this strain is adequate for catalyzing the decomposition of DMF. The specific properties of this unique Fe^3+^ site need further exploration, especially in the context of the reaction mechanism, where a very stable amide bond is hydrolytically cleaved.

## Conclusion

**Figure 4:**
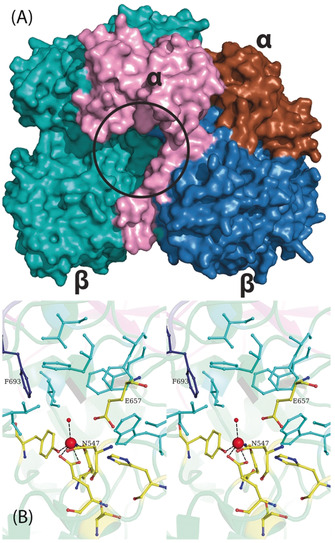
A) Surface representation of the dimer with the subunits colored differently. The entrance to the active site (black circle) is formed by the large and small subunits. B) A closer stereoview look at the binding pocket. The ligands to the Fe^+3^ and the residues involved in catalysis are labeled. The substrate‐binding site is made of a number of bulky aromatic residues depicted in cyan. Phe 693 from the loop of the neighboring large subunit is shown in dark blue.

The first biochemical characterization of a DMFase was reported in 1986, which indicated the possible presence of metal ion in the active site.^[14]^ The lack of any sequence or structural homology has hampered investigations into the active site of DMFases so far. The structure described here of DMFase from *Paracoccus* species strain DMF reveals two novel folds and an unusually coordinated iron as the active site. Furthermore, structure and biochemical experiments provide insight into residues involved in substrate binding and catalysis. A large cavity is observed at the interface between the large and small subunits, which leads towards the active site (Figure 4 A). In the EM structures of the active site mutants (Y440A and E521A), the interface between the large and the small subunit becomes disordered, thus suggesting a role for the small subunit in the substrate entry pathway (Figure S7). Bulky aromatic side chains flank the substrate‐binding pocket in the active site restricting access (Figure 4 B). The cavity formed by the hydrophobic residues is large enough to accommodate substitutions other than the dimethyl group on the amine. Currently, there is no structure available of the DMF‐bound form of the protein. Given the nature of the reaction, one would presume that the carbonyl center is oriented towards the active‐site iron, probably in coordination with the metal ion during the enzyme‐catalyzed reaction. The hydrophobic pocket may assist in the binding and orientation of the substrate, while the charged residues help in directing the carbonyl group toward the Fe^+3^.

Interestingly, phenylalanine (F693) from the adjacent large subunit is part of the substrate‐binding pocket (Figure 4 B). This side chain also acts as constriction and provides an explanation for selective substrate specificity and why larger amides are not efficiently hydrolyzed (Table 1). Cross‐talk between the two active sites in the dimer via the loop on which F693 is present is a distinct possibility. This residue and the loop may also be involved in the observed cooperativity in the kinetic experiments.

**Scheme 1:**
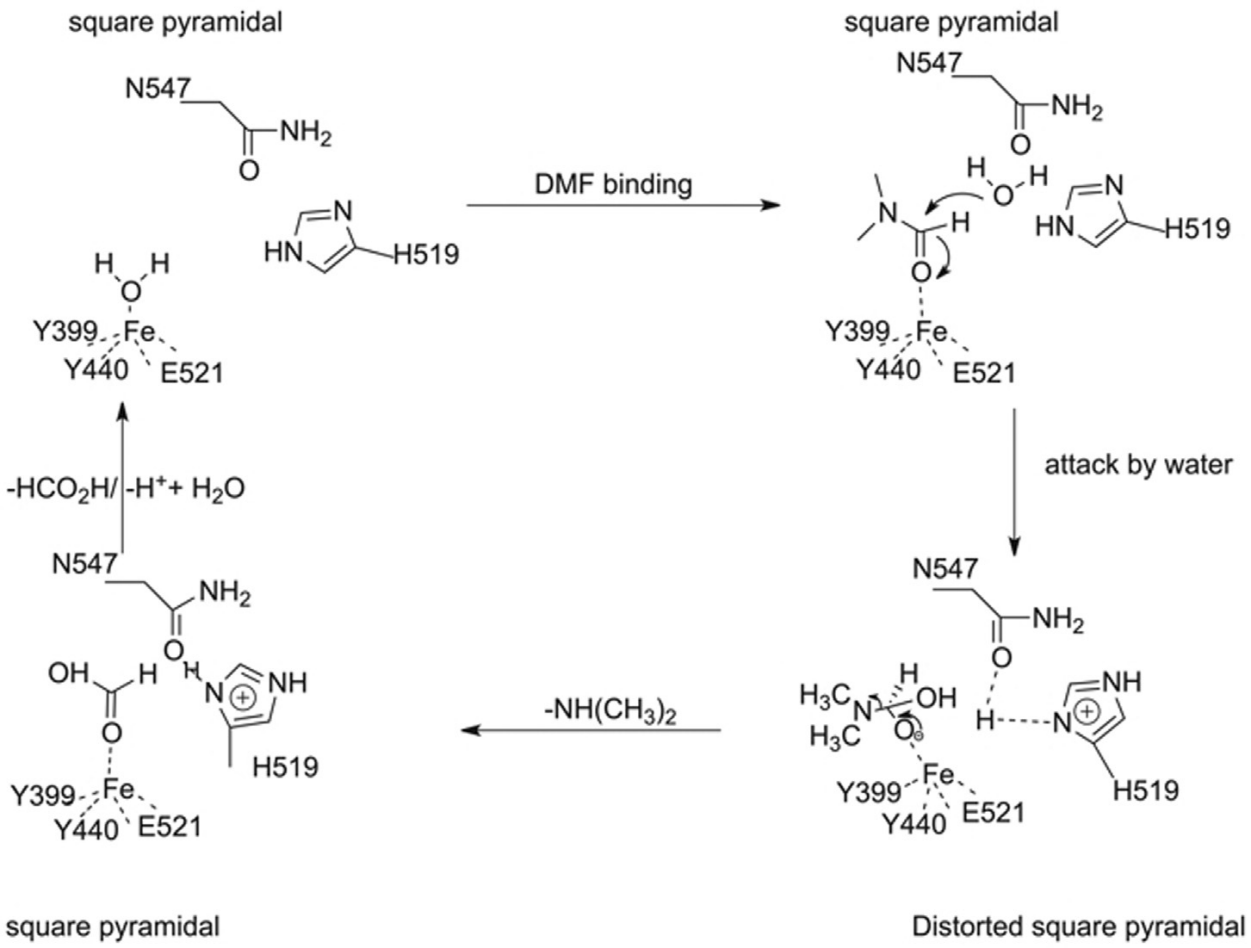
Proposed catalytic cycle for the parDMFase. Fe^III^ functions as a Lewis acid in this catalysis. All residues identified through mutagenesis are marked in this catalytic cycle.

The heat of formation of the peptide bond is 10.67 kJ mol^−1^, and that of an amide bond is 24.43 kJ per mole.^[22]^ The stability and properties that make DMF an attractive solvent would suggest that the molecule contains a very stable amide bond. The binding of water to Fe^3+^ would make it a better nucleophile to attack the C−N bond of the formamide. Our first hypothesis was that the binding of the C=O to the Fe^3+^ would also make the carbon a better electrophile, with no other residue being involved in catalysis. However, the observation of Glu657 close to the active site suggests that it might act as the catalytic base. One might consider a mechanism where the presence of the glutamate and the metal together provides the necessary catalytic power to break down the strong amide bond of DMF. We carried out site‐directed mutagenesis, and neither the E657A nor N547A mutants showed any catalytic activity against DMF. Together, one could propose a mechanism that involves the mononuclear Fe^3+^ center, Glu657, and the intermediate oxyanion stabilized by Asn547 (Figure 4 B; Scheme 1). In this case, the catalytic cycle must require water bound to Fe^3+^ as the ground state to which the substrate binds. The hydrolytic cleavage would then occur via a nucleophilic attack of the activated water on the predisposed amide bond of DMF, followed by the release of dimethylamine. Formate would then be displaced from the active site by the binding of another new water molecule, thereby ending the catalytic cycle.

While the evolutionary context of the new folds is not yet apparent, it is tempting to speculate as to why such a large size of the enzyme and a complex quaternary structure are required. These factors could help the catalyst with not only its halostability but also for stabilization of the strained Fe^+3^ site. This strained metal coordination, combined with the presence of a buried water molecule bound to N547, provides the necessary reduction in the activation energy for an increased rate of breakdown of the substrate. Further studies to elucidate the mechanistic details of this exciting reaction are in progress. Interestingly, the use of enzymes for bioremediation is often hindered due to a lack of stability of the catalyst in the purified form or the need for other partner proteins (often to provide the necessary electronic redox potential) for catalysis. The properties of the parDMFase described here suggest that this enzyme might be amenable for use in bioremediation with little enzyme engineering and without the need for additional proteins.

## Conflict of interest

The authors declare no conflict of interest.

## Supporting information

As a service to our authors and readers, this journal provides supporting information supplied by the authors. Such materials are peer reviewed and may be re‐organized for online delivery, but are not copy‐edited or typeset. Technical support issues arising from supporting information (other than missing files) should be addressed to the authors.

Supplementary
