# Supplementary material for: A 2‐Tyr‐1‐carboxylate Mononuclear Iron Center Forms the Active Site of a Paracoccus Dimethylformamidase
Source: Angew Chem Int Ed Engl. 2020 Jun 30;59(39):16961–6. doi: 10.1002/anie.202005332 (PMC7686228; doi:10.1002/anie.202005332)
Supplement: Supplementary file 1 — Supplementary [file ANIE-59-16961-s001.pdf]

Angewandte Chemie
Eine Zeitschrift der Gesellschaft Deutscher Chemiker

Supporting Information

A 2-Tyr-1-carboxylate Mononuclear Iron Center Forms the Active Site of a *Paracoccus* Dimethylformamidase

Chetan Kumar Arya, Swati Yadav, Jonathan Fine, Ana Casanal, Gaurav Chopra, Gurunath Ramanathan,* Kutti R. Vinothkumar,* and Ramaswamy Subramanian**

anie_202005332_sm_miscellaneous_information.pdf

Author Contributions

C.A. Data curation: Lead; Formal analysis: Lead; Investigation: Lead; Methodology: Equal; Writing—Original Draft: Equal; Writing—Review & Editing: Equal
S.Y. Formal analysis: Supporting; Investigation: Supporting
J.F. Formal analysis: Equal; Investigation: Equal
A.C. Data curation: Equal; Formal analysis: Equal
G.C. Data curation: Equal; Formal analysis: Equal; Funding acquisition: Equal; Supervision: Equal; Writing—Original Draft: Equal; Writing—Review & Editing: Equal
G.R. Conceptualization: Equal; Data curation: Equal; Formal analysis: Supporting; Funding acquisition: Equal; Supervision: Equal; Writing—Original Draft: Equal; Writing—Review & Editing: Equal
K.V. Data curation: Equal; Formal analysis: Equal; Investigation: Equal; Methodology: Equal; Supervision: Equal; Writing—Original Draft: Equal; Writing—Review & Editing: Equal
R.S. Conceptualization: Equal; Data curation: Equal; Formal analysis: Supporting; Funding acquisition: Lead; Investigation: Supporting; Methodology: Supporting; Project administration: Lead; Resources: Lead; Supervision: Equal; Validation: Equal; Visualization: Supporting; Writing—Original Draft: Equal; Writing—Review & Editing: Equal.

SUPPORTING INFORMATION

METHODS

Data reporting

No statistical methods were used to predetermine the sample size. The experiments were not randomized. The investigators were not blinded to allocation during the experiments and outcome assessment.

Bacterial strains and growth conditions

Strains used in this study were either *wild-type* and/or derived from chemically competent *E. coli* One Shot™ BL21 Star™ (DE3) (F^{-} *ompT hsdS*$_B$ *(rB*$^{-}$ *mB*$^{-}$*) gal dcm* (DE3)) (Invitrogen™). *wild-type(w.t.)* strain *Paracoccus sp.* strain DMF (NCBI BioSample accession no. SAMN11175380) was used to isolate and purify native parDMFase. Cells were grown aerobically at 37 ºC in an orbital shaker incubator (200 rpm) for 14 hours in modified minimal media. Compositions of modified minimal media are $Na_2HPO_4.2H_2O$ 2 g/L, KH_2PO_4 1 g/L, K_2SO_4 0.06 g/L, $MgSO_4$ 0.25 g/L, $CaCl_2.2H_2O$ 0.035 g/L, NaCl 0.5 g/L supplemented with DMF (1 g/L), yeast extract (0.01 g/L), and trace element solution (1 ml/L) {$Na_4EDTA.2H_2O$ 60 g/L, $ZnSO_4.7H_2O$ 22 g/L, $MnSO_4.H_2O$ 5 g/L, $CoCl_2.6H_2O$ 1.6 g/L, $CuSO_4.2H_2O$ 1.6 g/L, H_3BO_3 11 g/L, $(NH_4)_6Mo_7O_{24}.4H_2O$ 1.2 g/L, $NiSO_4.7H_2O$ 1.2 g/L and $FeSO_4.7H_2O$ 5 g/L (pH > 6 by adjusted by 10 N NaOH)}. For recombinant overexpression and purification of parDMFase, *E. coli.* cells were grown in LB media at 37 ºC.

Plasmid construction

Standard recombinant DNA techniques and In-Fusion® HD Cloning (Takara Bio USA, Inc.) were used for plasmid construction in this study and were verified by DNA sequencing. The Plasmid pET-21a (+) (*lacI*, ori F1, ori pBR322, T7 promoter and terminator, Amp^R) was used to generate the *E. coli* overexpression vectors harboring parDMFase (*w.t.*), parDMFase (β2), parDMFase (S395A), parDMFase (Y399A), parDMFase (Y440A), parDMFase (H519A), and parDMFase (E521A) with a C-terminal 6x-His tag for protein purification. The gene encoding for parDMFase was generated by PCR amplification of *dmfase* gene using a bacterial plasmid isolated from *Paracoccus sp.* strain DMF as template DNA. The spin-column purified PCR product was ligated with linearized pET21(a)+ vector at *NdeI* and *HindIII* cloning sites and transformed into One Shot™ TOP10™chemically competent *E. coli* (Invitrogen™) for positive clone screening and plasmid isolation.

Protein expression and purification

parDMFase was purified from both the *wild-type* and recombination sources. Cell pellets of *Paracoccus sp.* strain DMF were washed twice with **buffer A** (Tris-Cl 50 mM, NaCl 50 mM, dithiothreitol (DTT) 1 mM, pH 7.2 at 4 ºC) and re-suspended in cell **lysis buffer B** (buffer A, NaCl 200 mM, $MgCl_2$ 10 mM, lysozyme 0.3 mg/mL, DNase 0.01 mg/mL, glycerol 10% v/v) and incubated at 4 °C for a minimum of 4h before lysis by sonication. The cell extract was obtained by centrifugation of the lysate at 20000*g for 45 min at 4 ºC. Purification was achieved by high-salt precipitation followed by ion-exchange chromatography. Ammonium sulfate salt was added to the cell extract culture (50-70% saturation range). The resulting precipitate was collected by centrifugation at 10,000*g for 10 min and re-dissolved in buffer A and dialyzed overnight against three exchanges of the same buffer. The dialyzed protein solution was incubated at 45 °C for 15 min on a heating block and was immediately transferred into ice, and heat-denatured aggregated protein fractions were removed by centrifugation at 12000*g for 15 min at 4 °C. Two-step anion-exchange chromatography was performed for further purification. The protein solution was injected into a HiPrep Q-FF (16/10) column (GE Healthcare Life Sciences) pre-equilibrated with buffer A. Column was washed with 10 CV buffer containing 0.2 M NaCl and protein was eluted with 20 CV of the same buffer with a linear gradient of 0.2 to 0.6 M NaCl. A flow rate of 1 mL/min was maintained throughout the purification process. The purified fractions as determined by SDS-PAGE (12%) were pooled and concentrated using a Millipore Amicon ultra 100K device. Dialysis was performed for 6h against buffer A and re-injected into the same column. Protein was eluted on a linear gradient of NaCl (0.3 to 0.6 M). This step was found to be essential for purification of the native parDMFase to homogeneity. Peak fractions from the anion-exchange column were pooled, concentrated, and was followed by size exclusion chromatography (SEC) with a Superdex column (S-200, 16/60, GE Life Sciences) pre-equilibrated with buffer C (Tris-Cl 50 mM, NaCl 250 mM, DTT 1 mM, pH 7.2 at 4 ºC). The parDMFase was eluted with the same buffer at the flow rate of 1 mL/min. All the peak and pooled fractions were tested for enzymatic activity.

The parDMFase constructs fused to a C-terminal His_6 tag were expressed in *E. coli* BL21 star (DE3) cells. Cells were grown in LB medium supplemented with ampicillin (100 µg/ml) at 37 °C until they reached an OD_{600nm} of ~0.6. Protein overexpression was induced by the addition of 0.5 mM IPTG and cultures were grown for 6h at 37°C. Cell pellets were pooled by centrifugation at 4000*g for 15 min, and resuspended in lysis buffer B and sonicated. The lysate was cleared by centrifugation at 14000*g for 45 min at 4°C. Further purification of the recombinant enzyme was performed using steps as described for native enzyme purification. SEC elution fractions

corresponding to parDMFase were either used immediately or flash frozen in liquid nitrogen with 20% glycerol and stored at -80 °C until further use.

Protein concentration determination

Protein concentration at each purification step was estimated by the Bradford method using bovine serum albumin (BSA) as a standard[1].

Steady-state enzyme assay

Amidohydrolase activity of parDMFase towards *N*-substituted amides was determined using an alkylamine-specific colorimetric assay (Cullis and Waddington 1956). The standard enzyme assay with DMF contained 45 µL of 50 mM buffer C, 50 µL enzyme solution, and 5 µL of 3M DMF. The reaction was carried out in tightly closed microcentrifuge tubes for 30 min at 37°C. The reaction was quenched by the addition of 25 µL trichloroacetic acid (TCA) solution (15%, w/v). After centrifugation at 10000*g for 20 min, 100 µL of the reaction mixture was added to 1 mL of carbonate buffer (sodium tetraborate hexahydrate (9.53 g/L) and sodium carbonate (5.3 g/L) solution (pH 9.8)), 0.25 mL freshly prepared sodium nitroprusside ($Na_2[Fe(CN)_5NO]$) solution (1% w/v), and 0.25 mL acetaldehyde solution (l0% v/v). The dimethylamine (DMA) formed in the enzymatic reaction leads to a change in the absorbance, which was measured after 20 min at 580 nm. Spectrophotometric measurements were carried out with a blank solution in which DMF was added to the enzyme mixture after the enzyme inactivation step with TCA. The concentration of DMA produced upon enzymatic reaction was determined from a standard curve calibrated with known concentrations of DMA.

Isothermal Titration Calorimetric (ITC) enzyme assay

To perform the real-time ITC enzyme kinetic assay, two sets of experiments were carried out. In single injection mode, the total molar enthalpy (ΔH^o) was extrapolated by titrating 25 µL from a single injection of a relatively low concentration of substrate (25 mM) to a relatively high concentration (25 nM, 203.7 µL) of parDMFase in the cell (Microcal ITC200, GE-Healthcare). In the multiple injection mode, enzymatic reaction was initiated by a series of injections of a relatively high concentration of DMF (100 mM, 2 µL, 20 injections) with a relatively low concentration of enzyme (25 nM) in the cell. All experiments were performed in buffer C at 37 °C in high-feedback mode with a stirring speed of 1000 rotations per minute and a filter time of 4s. A pre-injection delay of 600 s was applied in order to establish a steady baseline. A plot of heat rate *vs*. substrate concentration points was obtained in order to calculate the kinetic parameters after a baseline

correction. The ΔH^o value was determined in terms of the shift in thermal power that occurred from the conversion of the substrate before returning to its pre-equilibrated base-line[2]. The Michaelis-Menten enzyme parameters K_M, k_{cat}, and steady-state heat (pseudo first order) rate were derived from the enzyme that was titrated with increasing concentrations of substrate. The kinetic experiments were carried out in duplicate. A non-linear regression fitting the Michaelis-Menten equation (GraphPad Prism v6.0 software) was used to calculate the kinetic parameters (K_m and k_{cat}).

^{1}H-NMR spectroscopy of enzyme kinetics for substrate specificity

Hydrolase activity of parDMFase towards DMF substrate analogs including formamide, acetamide, *N*-methylformamide (NMF), *N*-ethylformamide (NEF), dimethylacetamide (DMAc), diethylformamide (DEF), benzamide, hexanamide, and urea were examined using real time ^{1}H-NMR based kinetic assays. NMR experiments were carried out using Avance 600 MHz NMR spectrometer in a 5 mm probe at 310 K. All samples were prepared in 25 mM sodium phosphate buffer (pH 7.2) containing 250 mM NaCl. For the kinetics experiment, 10 nM parDMFase was added to a reaction mix (500 µL) containing various concentrations of DMF and 1 mM α-alanine (internal standard). Stock solutions of DMF and α-alanine were prepared in a D_2O assay buffer consisting of 20 mM phosphate buffer (4:1), 250mM NaCl, pH 7.2, at 37 °C. The rate of the reaction was obtained by calculating the rate of disappearance of the carbonyl peak –CH(O) of the substrate. The initial and final concentrations of the substrates were calculated by integrating the peak area of the carbonyl signal of amide with respect to the acetyl ($-CH_3$) signal of α-alanine as an internal standard. The kinetic experiments were carried out in duplicate. A non-linear regression fitting the Michaelis-Menten equation (GraphPad Prism version 6.0 software) was used to calculate the kinetic parameters (K_M and k_{cat}).

Thermal shift assay

Protein unfolding and stability were determined as a function of temperature. In experimental studies, a label-free thermal shift assay was performed using Tycho NT. 6 (NanoTemper Technologies). Pre-dialyzed protein and mutant samples were diluted (~0.5 mg/mL) in appropriate buffer conditions and run in duplicates in capillary tubes. Intrinsic fluorescence from tryptophan and tyrosine residues was recorded at 330 nm and 350 nm while heating the sample from 35°C to 95°C at a ramp rate of 30°C/min. The ratio of fluorescence (350/330 nm) and the inflection temperature (T_i) were calculated using Tycho NT. 6 software, which provides T_m (melting point), the temperature at which 50% of the measured protein is unfolded.

Electron microscopy of parDMFase

Initial images of parDMFase data at 2-3 mg/ml in 50 mM NaCl were collected using Quantifoil holey carbon grids (R 0.6/1, Au 300 mesh) with blotting and freezing accomplished with a manual plunger in a cold room. This initial data was collected with Titan Krios at MRC LMB, Cambridge, and Falcon 2 detector in integration mode with the EPU software. Images showed that there were two populations, and both these were picked and subjected to reference-free 2D classification. Initial models of both the populations were individually generated either with EMAN2[3] or by Stochastic gradient descent within RELION[4] with C2 symmetry imposed. The refinement of the dimer population obtained in the integration mode gave a reconstruction of ~7 Å, indicating that the protein behaved well and a high resolution structure can be obtained. All the data described here were collected from the enzyme obtained by recombinant expression. Enzyme purified from native source showed similar maps (data not shown).

Subsequently, data were collected with a Falcon 3 detector in counting mode at 1.07 Å sampling and images were exposed for 60 seconds with a total accumulated dose of ~27 e/Å^2 and dose fractionated into 75 frames, with each frame having a dose ~0.3 e. The images were grouped into 25 frames, resulting in ~1 e/frame, and Unblur [5] was used for alignment. This initial processing was performed in Relion 2.0 [6]. The summed images were then used for automated particle picking with Gautomatch with template derived from previous data collection, and CTF was estimated with Gctf[7]. Particles were extracted with a box size of 320 pixels and subjected to 2D classification, 3D auto-refinement, per particle motion-correction, B-factor weighting, and refinement. Further 3D classification was used to improve the quality of the maps by removing bad particles. This resulted in a 3.4 Å map for the dimer and 3.8 Å for tetramer. We noticed that the views in the tetramer were not diverse, may be because of thinner ice.

During this period, we observed that the oligomeric state of the enzyme is affected by salt and subsequently we imaged parDMFase in three different salt concentrations (no salt, 200 and 500 mM NaCl). All of the data presented here were collected at the National CryoEM facility in Bangalore with a Falcon 3 detector in counting mode at 1.07 Å sampling and 60s exposure. The grids for these datasets were Au 300 mesh either R0.6/1 or 1.2/1.3 and made with Vitrobot Mark IV at 100% relative humidity and 16°C. The grids were blotted for 3.5 seconds. The datasets were processed with Relion 3.0, including the whole frame alignment and dose-weighting with Relion's own algorithm. Particle picking and CTF estimation were performed using Gautomatch and Gctf. Particles were extracted with a box size of 320 pixels and subjected to 2D classification, 3D auto-

refinement, per particle CTF refinement, B-factor weighting with Bayesian polishing and refinement, and subsequent 3D classification [8]. The data sets of mutants Y440A and E521A were processed similarly. The local resolution of the maps was estimated using Resmap[9]. Model building was performed with Coot[10], and the model was refined with phenix.real_space_refine[11]. Although there are non-protein densities in the map, we modelled only one water molecule at the active site and the rest were not modelled. Details of the EM data and model quality are presented in Supporting information Table 2.

Crystallization, refinement and model building

The purified native and recombinant parDMFase were concentrated to 10 and 5 mg ml^{-1}. Initial crystallization trials for the purified parDMFase were carried out using the commercially available screens PEG/Ion, PEG/Ion 2, Crystal Screen, Crystal Screen 2 (Hampton Research) and Wizard Classic 1 and 2 (Rigaku). Crystals of parDMFase were obtained by mixing 200 nl of protein in buffer A with equal volumes of precipitant. All trials were conducted by hanging-drop vapour diffusion and incubated at 18°C. X-ray diffracting protein crystals were obtained in a condition consisting of 0.1 M Tris pH 7.5, 0.2 M KCl, 18% w/v PEG 3350 after refinement of the screening conditions. The crystals were cryoprotected with reservoir buffer containing 10% ethylene glycol and flash-frozen in liquid nitrogen (*liq*. N_2). Data were collected from single crystals at Advanced Light Source (ALS) Berkeley and subsequently scaled and reduced with XDS. Preliminary phasing of parDMFase crystals diffracted to around 3.5 Å was performed by molecular replacement in Phaser (Phenix) using a search model derived from EM model generated above mentioned. No molecular replacement solution with found. The observation in cryoEM that two oligomeric state of parDMFase exist in solution depending on the salt concentration prompted us to set up crystallization with enzyme purified in presence of high salt concentration. This yielded highly reproducible crystals that diffracted up to 2.9 Å. The crystals belonged to space group $P2_1$ and molecular replacement using the tetramer model obtained from the EM map a solution was obtained. Iterative cycles of model rebuilding and refinement were performed in COOT and Phenix.refine respectively. Structure validation with wwPDB and PDBREDO were integrated into the iterative rebuild and refinement process. There are two tetramer in the asymmetric unit and the model was refined against the data with non-crystallographic symmetry. The final R-factor and R-free of that reflect the fit of the model to the structure are 0.23 and 0.27 respectively. Coordinates and structure factors have been deposited in the Protein Data Bank (PDB) under accession numbers 6LVV.

Synchrotron X-ray fluorescence spectroscopy (syncXRF)

The identity of the metal present in parDMFase was validated by XRF of protein crystals. Protein crystals were flash frozen in liquid N_2, and sequentially washed four times in the cryoprotectant consisting of 9:1 v/v of mother liquor and ethylene glycol. XRF scans were taken at the PROXIMA-1 beamline at the SOLEIL synchrotron at 107K. The spectrum contains X-ray emission lines characteristic of Fe. The peak at ~7,100 eV corresponds to the K_α emission energy of iron.

Electron Paramagnetic Resonance (EPR) study of parDMFase

Protein samples for EPR were prepared by concentrating the protein up to 20 mM in buffer A. Samples were taken in the probe and frozen in liquid nitrogen. EPR spectra were obtained on a Bruker EMX EPR spectrometer at IIT Kanpur. Spectra processing and simulation were performed using a Bruker WIN-EPR and SimFonia software. A control experiment with buffer was also carried out.

Salt, temperature, and solvent dependent parDMFase stability and activity

To understand the effect of temperature and salt concentration on the activity and stability of parDMFase, we performed steady-state enzyme assays, and thermal shift assays in various assay conditions. All experiments were performed in sodium phosphate buffer (25 mM, pH 7.2, NaCl 50 mM, DTT 1 mM) and in triplicate. For thermal shift measurements, enzyme at different salt concentrations, parDMFase (600 nM) were first incubated for 24 hours at room temperature in phosphate buffer containing increasing NaCl concentrations ranging from 50-5000 mM. the inflection temperature (T_i) of each sample was obtained by thermal shift assay performed on a Tycho NT.6 system. Next, we performed steady-state enzyme kinetics experiments to determine the relative activity of parDMFase towards DMF. The measurements were carried out in 50 μL reaction mixture, containing final concentrations of enzyme (600 nM) and DMF (300 mM) at 37°C for 20 min. Enzymatic reaction was inhibited by protein denaturation by adding TCA, and further a colorimetric assay was performed to determine the DMA concentration as described above. Similarly, the effect of temperature on enzyme activity was measured by performing steady-state enzyme assay at different temperatures and measuring the relative catalytic activity with reference to the activity at 37°C.

To understand the solvent-based stability of parDMFase, parDMFase was incubated for at least 24h in different DMF-buffer concentrations at 4°C. The structural integrity of parDMFase was measured by thermal unfolding profile using a Tycho 1.6. The enzymatic response of incubates

was recorded by a time-independent assay at 37°C. The thermal unfolding characterization of incubates shows a gradual decay in mean T_i with an increased organic-aqueous ratio. Interestingly, DMF showed a stabilization effect (with increased T_i of 3°C) up to ~7.5% v/v solvent content. A higher organic -- aqueous ratio leads to conformational instability by monitoring the blue shifts in the detected first inflection temperature. The loss of the native thermal unfolding profile occurs after 65% v/v solvent presence (>7M). Enzyme catalytic response showed gradual decay of activity as opposed to a sharp decline in higher solvent medium. Optimum relative activity measured for DMF was at 3.1% v/v. In high organic solvent medium (>40%), disintegration of the catalytic structure leads to the loss of total activity even with the remaining residual quaternary structure.

Density functional theory calculations for strained iron in par***DMFase***

We used a recently published method for calculating the electronic energies of the iron complex using density functional theory[12] with the B3LYP functional for singlet calculations, the UB3LYP method for doublet calculations, and the 6-311G(d) basis set. We used the 6-311G(d) basis set for all atoms, as the difference in computation time was not significant. We assumed a low spin state of iron in both octahedral and tetrahedral calculations. The Fe^{3+} charge state results in a doublet, and the Fe^{2+} charge state results in a singlet regardless of the tetrahedral or octahedral symmetry around the iron atom. Moreover, both tyrosine residues were assumed to be deprotonated, along with the glutamic acid residue. Therefore, the Fe^{3+} case results in a neutral system, whereas the Fe^{2+} case results in an anionic state. The initial coordinates were obtained from the crystal structure and the beta carbon was replaced with a methyl group to sever as a single bond between two SP^3 carbons. For each geometry and charge state, two optimizations were performed: the first was constrained so that only the hydrogens and water atoms were allowed to move (strained state), and a second optimization was were all atoms were allowed to move (relaxed state). For the doublet calculations, where an unrestricted calculation was performed, the $\langle S^2 \rangle$ values were calculated before and after the annihilation of the first spin contaminant (Supporting information Table 3). The expected value of $\langle S^2 \rangle$ for the system lacking any spin contamination is 0.75 for the doublet system. Since $\langle S^2 \rangle$ after spin annihilation for both Fe^{3+} symmetries are 0.7559 and 0.7503 (Supporting information Table 3), which is relatively close to 0.75, we conclude that our calculations do not suffer from spin contamination. All calculations were performed using Gaussian16 (Frisch, M. J. *et al.* Gaussian16 Revision B.01. (2016)), and the geometries of the octahedral case for both optimizations are provided in Supporting information Figure 8**.** For the calculation of the acetal intermediate of DMF, the B3LYP functional was used with the 6-311G(d) basis set. The initial coordinates of DMF and the transition state of the reaction, water, and acetal

were obtained using the build molecule functionality of GaussView 6.0. All structures were checked for negative frequencies to ensure that a proper intermediate was found.

Molecular Figures were made with Pymol[13] or Chimera[14].

Data availability: The EMDB and PDB codes for the structures described include: tetramer (EMDB-0988, 6LVB), dimer (EMBD-0989, 6LVC), Y440A mutant (EMDB-0990, 6LVD), E521A mutant (EMD-0991, 6LVE). The structure factors and coordinates of the X-ray data have been deposited with a PDB code 6LVV.

REFERENCES:

[1] M. Bradford, *Anal. Biochem.* **1976**, *72*, 248–254.
[2] L. D. Hansen, M. K. Transtrum, C. Quinn, N. Demarse, *Biochim. Biophys. Acta - Gen. Subj.* **2016**, *1860*, 957–966.
[3] G. Tang, L. Peng, P. R. Baldwin, D. S. Mann, W. Jiang, I. Rees, S. J. Ludtke, *J. Struct. Biol.* **2007**, *157*, 38–46.
[4] D. Kimanius, B. O. Forsberg, S. H. Scheres, E. Lindahl, *Elife* **2016**, *5*, DOI 10.7554/eLife.18722.
[5] A. F. Brilot, J. Z. Chen, A. Cheng, J. Pan, S. C. Harrison, C. S. Potter, B. Carragher, R. Henderson, N. Grigorieff, *J. Struct. Biol.* **2012**, *177*, 630–637.
[6] S. H. W. Scheres, *J. Struct. Biol.* **2012**, *180*, DOI 10.1016/j.jsb.2012.09.006.
[7] K. Zhang, *J. Struct. Biol.* **2016**, *193*, 1–12.
[8] J. Zivanov, T. Nakane, B. O. Forsberg, D. Kimanius, W. J. H. Hagen, E. Lindahl, S. H. W. Scheres, *Elife* **2018**, *7*, DOI 10.7554/eLife.42166.
[9] T Kucukelbir A, Sigworth FJ, Tagare HD. Quantifying the local resolution of cryo-EM density maps. *Nat Methods*. 2014;11(1):63-65. doi:10.1038/nmeth.2727
[10] P. Emsley, B. Lohkamp, W. G. Scott, K. Cowtan, *Acta Crystallogr. D. Biol. Crystallogr.* **2010**, *66*, 486–501.
[11] P. D. Adams, P. V Afonine, G. Bunkóczi, V. B. Chen, N. Echols, J. J. Headd, L.-W. Hung, S. Jain, G. J. Kapral, R. W. Grosse Kunstleve, et al., *Methods* **2011**, *55*, 94–106.
[12] Y. I. A. Reyes, F. C. Franco, *Phys. Chem. Chem. Phys.* **2019**, *21*, 16515–16525.
[13] W. L. DeLano, **2000**.
[14] E. F. Pettersen, T. D. Goddard, C. C. Huang, G. S. Couch, D. M. Greenblatt, E. C. Meng, T. E. Ferrin, *J. Comput. Chem.* **2004**, DOI 10.1002/jcc.20084.

Supporting information Table 1. Real time kinetic parameters of parDMFase at 37ºC

Enzyme	**par**DMFase (nM)	DMF (mM)	ΔH (kcal/mol)	k_{cat} (s^{-1})	K_m (mM)	V_{max}	k_{cat}/K_m
Native **par**DMFase	25	100	-1.4	115 (±1.5e^{-6})	0.97 (±0.06)	2.89	118
Recombinant **par**DMFase	25	100	-1.6	202 (±2.0e^{-6})	0.87 (±0.07)	5.13	232

Supporting information Table 2. Cryo-EM Data collection and refinement statistics for parDMFase.

	parDMFase 200 kDa, $\alpha_2\beta_2$	parDMFase 400 kDa, 2x($\alpha_2\beta_2$)	parDMFase 400 kDa, 2x($\alpha_2\beta_2$), Y440A mutant	parDMFase 400 kDa, 2x($\alpha_2\beta_2$), E521A mutant
Data Collection				
Pixel Size (Å)	1.07	1.07	1.07	1.07
Dose rate (e/p/s)	0.52	0.52	0.56	0.56
Total dose (e/Å^2)	~27	~27	~27.5	~27.5
No. of particles	80674	59191	18645	45851
Resolution (nominal), Å	3.0	2.8	3.2	3.1
B-factor (sharpening)	95	86	95	92
Model composition				
Number of chains	4	8	8	8
Protein (No. of residues)	1772	3544	3464	3480
Ligand	2	4	0	0
Model Refinement				
Resolution (Å)	3.0	2.8	3.2	3.1
Average B-factor				
Overall	43.1	38.8	51.5	50.7
Protein	43.1	38.8	51.5	50.7
Ligand (Fe)	46.3	43.5	-	-
RMS deviations				
Bonds (Å)	0.01	0.009	0.009	0.01
Angles (Å)	1.3	1.2	1.2	1.3
Validation				
Ramachandram plot (favoured/outliers)	94.2/0.0	95.4/0	93.9/0	93.8/0
Clashscore	3.9	2.9	3.4	3.3

Molprobity score	1.6	1.4	1.5	1.5

Supporting information Table 3. Electronic energies of the iron's electronic environment. All values in atomic units unless otherwise noted. The <S^2> values before and after the annihilation of the first spin contaminate are separate with a '/'.

Optimization	Fe^{3+} Tetrahedral (Doublet)	Fe^{3+} Octahedral (Doublet)	Fe^{2+} Tetrahedral (Singlet)	Fe^{2+} Octahedral (Singlet)
**Hydrogens and water flexible, strained state (Hartree)**	-2261.2005	-2337.6695	-2261.2406	-2337.7092
**All atoms flexible, relaxed state (Hartree)**	-2261.2843	-2337.7540	-2261.3356	-2337.8059
**Difference (Hartree)**	-0.083848	-0.084562	-0.095001	-0.096712
**Difference (kJ/mol)**	-220.1429	-222.0175	-249.4251	-253.9174
**Spin contamination**	1.0345 / 0.7559	0.7811 / 0.7503	N/A	N/A

Supporting information Table 4. Electronic and Gibbs free energies of the gas phase reaction between DMF and water.

	Electronic (Hartrees)	Gibbs (Hartrees)	Electronic difference with reactants (kJ/mol)	Gibbs difference with reactants (kJ/mol)
**Reactants**	-325.0080664	-324.93114	0	0
**TS**	-324.948649	-324.856166	155.9980879	196.841238
**Acetal Product**	-324.9976268	-324.897909	27.40884096	87.24666126

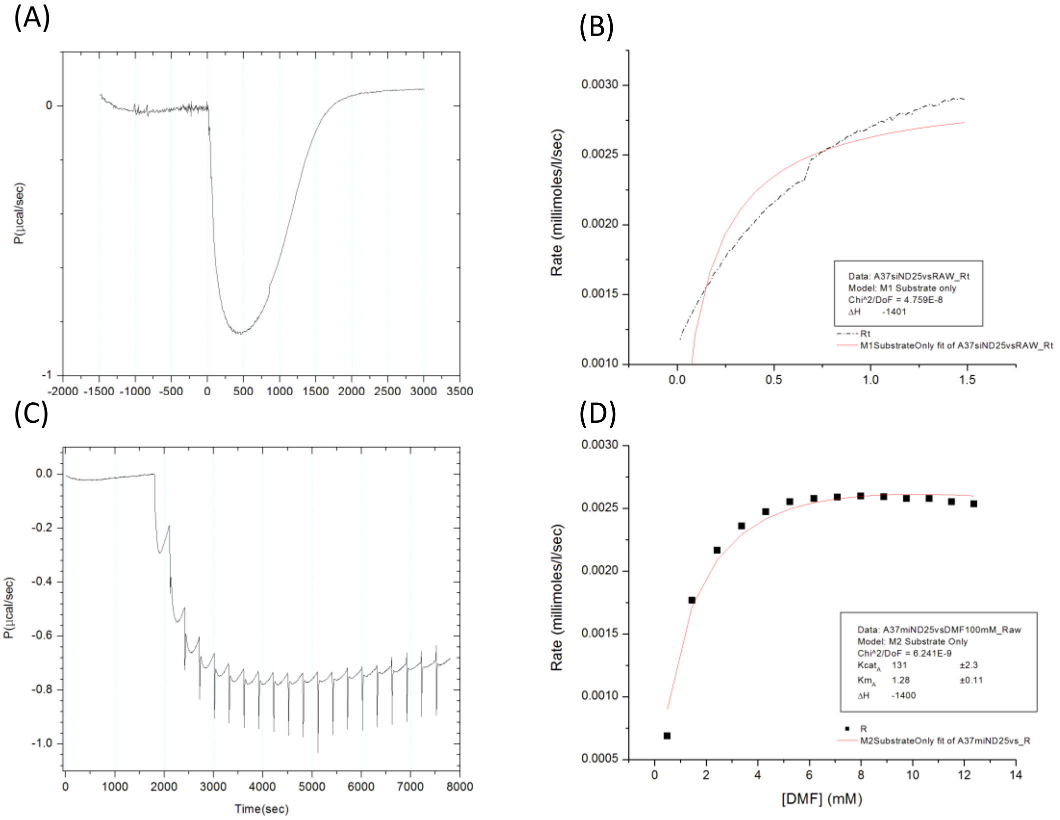

Supporting information Figure 1: ITC kinetic assay for native parDMFase catalyzed hydrolysis. (A) Raw data for thermal power (μcal/sec) single injection thermographs at 37ºC. (B) Data from (A) has been transformed into reaction rates vs. substrate concentration [S] (mM) with the nonlinear least squares fit to the Michaelis-Menten equation for ΔH determination. Calorimetric assay thermograph; (C) Raw data from Multiple injection method for thermal power (μcal/sec) at 37ºC. (D) K_m and k_{cat} with the non-linear least squares fit to the Hill equation from plot rate vs. [DMF].

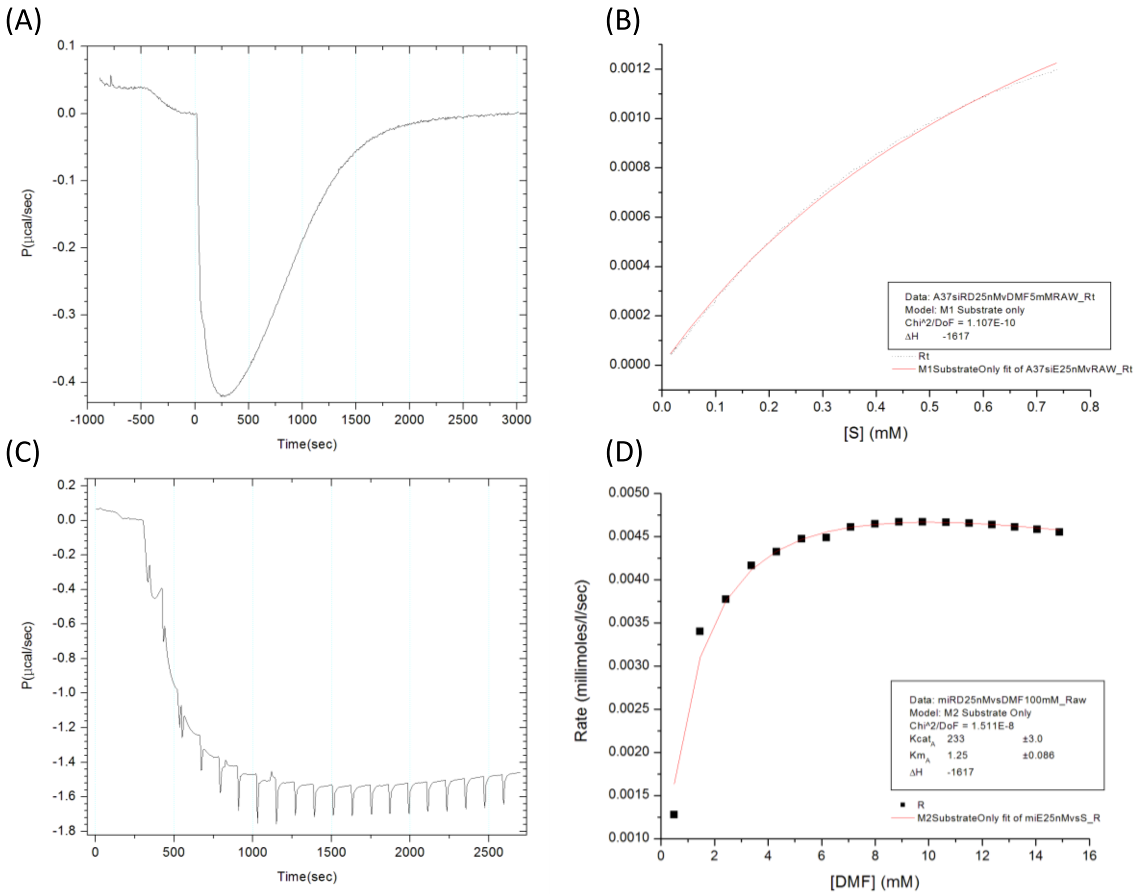

Supporting information Figure 2: Normalized ITC single injection thermographs for recombinant parDMFase catalyzed hydrolysis. (A) Raw data for thermal power (µcal/sec) at 37ºC. (B) Data from (A) has been transformed into reaction rates (millimoles/I/sec) vs. substrate with the nonlinear least squares fit to the Michaelis–Menten equation for ΔH determination. Calorimetric assay thermograph; (C) Raw data from Multiple injection method for thermal power (µcal/sec) at 37ºC. (D) K_M and k_{cat} with the non-linear least squares fit to the Hill equation from plot rate vs. [DMF].

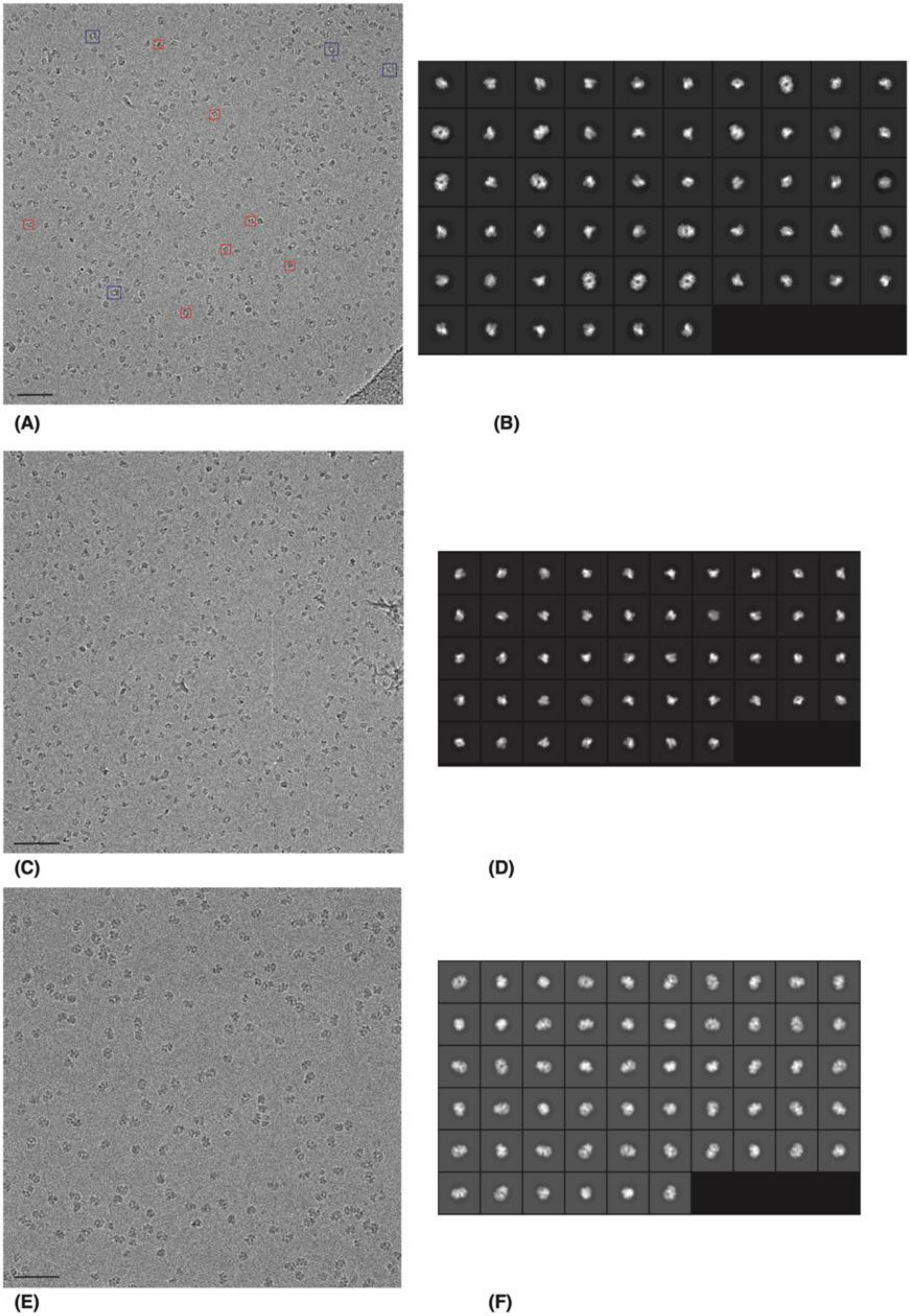
(A)
(B)
(C)
(D)
(E)
(F)

Supporting information Figure 3: CryoEM micrographs and 2D classifications

(A) Micrograph of parDMFase that shows mixed population. The smaller molecule (shown in red box) and larger molecule (blue box) are observed. Scale bar is 500 Å.

(B) All the particles from mixed populations was picked and subjected to reference-free 2D classification, both the smaller and large molecule could be separated. Box size is 256 pixels and sampled at 1.38 Å/pixel.

(C) parDMFase imaged with no salt in the buffer. This shows predominantly dimer population. Scale bar is 500 Å.

(D) Representative reference-free 2D class averages of the molecule with no salt buffer. Box size is 320 pixels and sampled at 1.07 Å/pixel.

(E) parDMFase imaged with 200 mM of NaCl in the buffer. This shows predominantly tetramer population. Scale bar is 500 Å.

(F) Representative reference-free 2D class averages of the molecule with 200mM. Box size is 320 pixels and sampled at 1.07 Å/pixel.

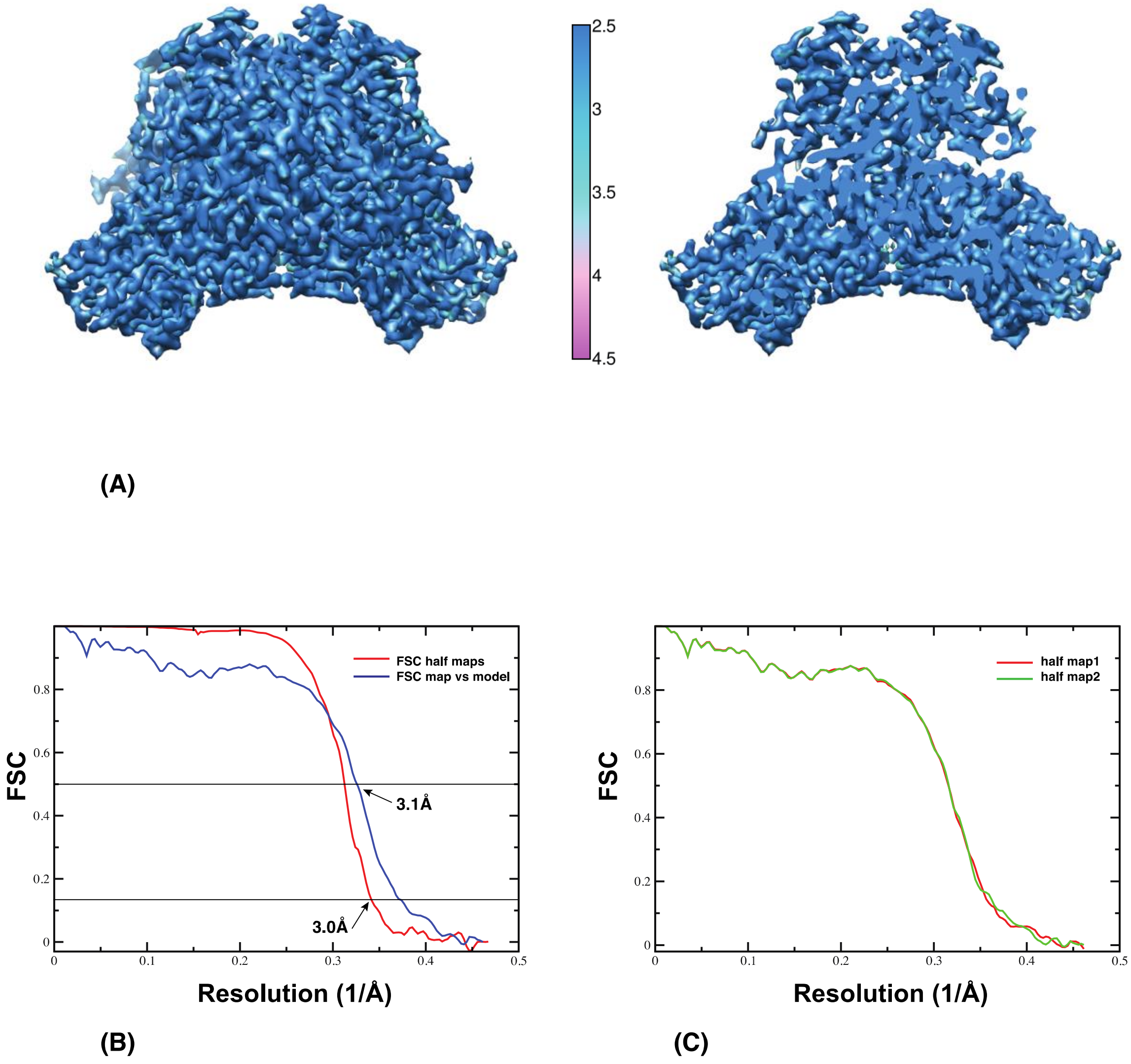

Supporting information Figure 4: Local resolution of the parDMFase dimer and the FSC plots

(A) The local resolution plot of parDMFase dimer showing the whole molecule on left and a cut away section on right. Much of the molecule shows resolution between 2.5-3.5 Å allowing the building of the complete model.

(B) The Fourier shell correlation (FSC) curves of two half-maps (red) and the map and model (blue) are shown.

(C) To verify overfitting of the model refinement, one of the half-map used in refinement and the other half-map was used as test.

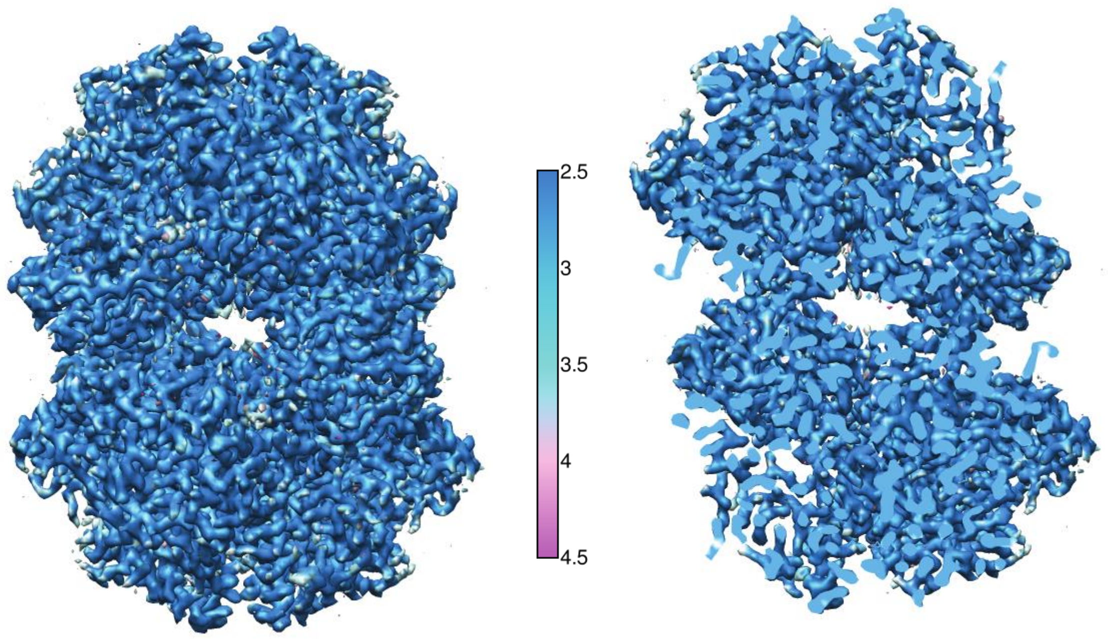

(A)

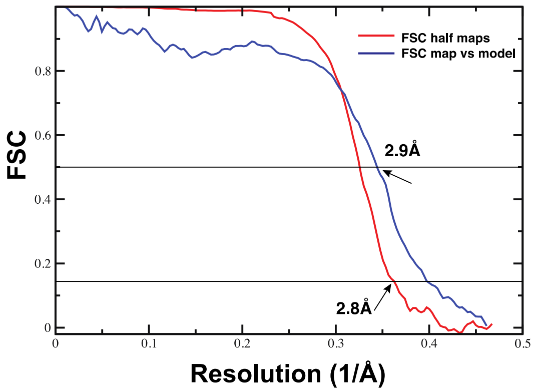

(B)

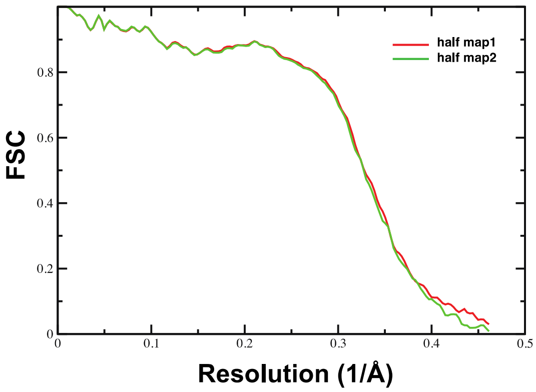

(C)

Supporting information Figure 5: Local resolution of the parDMFase tetramer and the FSC plots

(A)The local resolution plot of parDMFase tetramer showing the whole molecule on left and a cut away section on right. Much of the molecule shows resolution between 2.5-3.5 Å allowing the building of the complete model.

(B)The Fourier shell correlation (FSC) curves of two half-maps (red) and the map and model (blue) are shown.

(C) To verify overfitting of the model refinement, one of the half-map used in refinement and the other half-map was used as test.

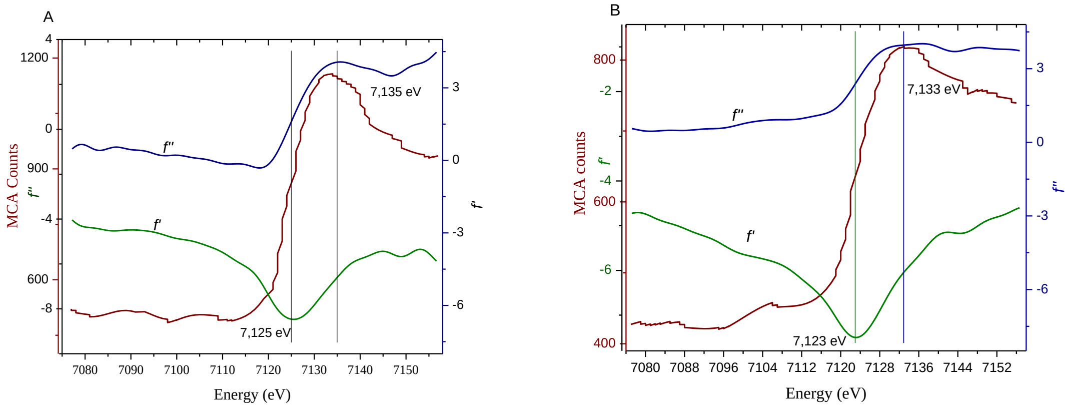

Supporting information Figure 6A: X-ray fluorescence scan of (A) native and (B) recombinant parDMFase crystals at the Iron K-edge region. The *f''* plot and the observed absorption edge (7,135 eV or 2.10 Å) correspond to values for the iron K_α edge.

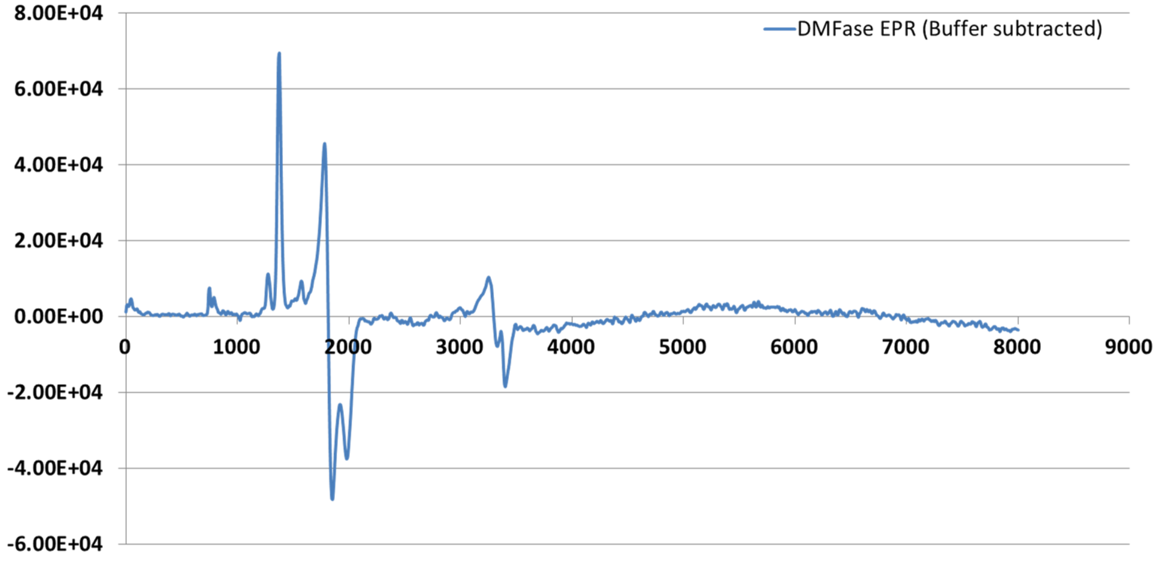

Supporting information Figure 6B: Buffer subtracted EPR spectra of 100µM parDMFase in a buffer containing 20mM HEPES, 200mM NaCl, 1mM DTT and 10% glycerol is shown. EPR spectra in the figure was recorded using Bruker ELEXSYS-IIX-band (ν = 9.458 GHz) digital EPR spectrometer. The measurement was done at liquid nitrogen temperature (77 K).

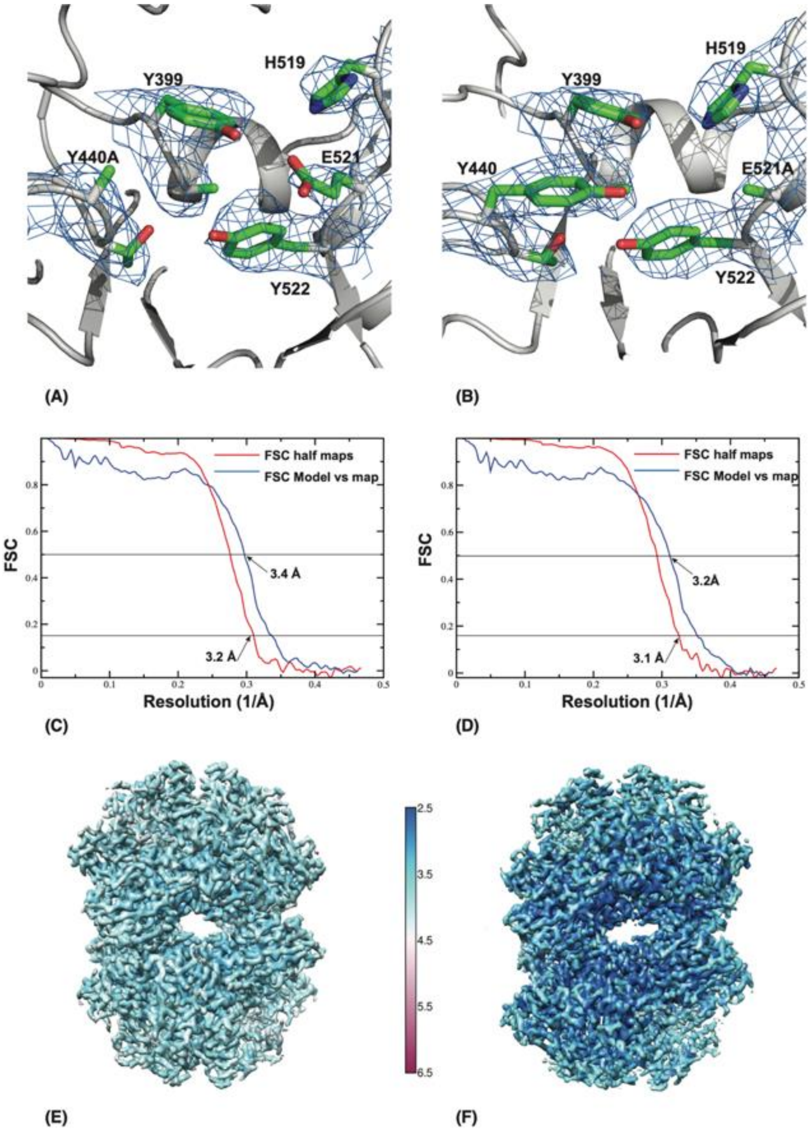

Supporting information Figure 7: Cryo-EM structures of Y440A and E521A

(A & B) The active site of the Y440A and E521A mutants. Density around the active site residues that coordinate Fe are shown. In both these mutants, the extra density that is seen in wild type enzyme is absent.

(C & D) The Fourier shell correlation (FSC) curves of two half-maps (red) and the map and model (blue) are shown for parDMFase Y440A and E521A mutants respectively.

(E & F)The local resolution plots of parDMFase Y440A and E521A mutant respectively.

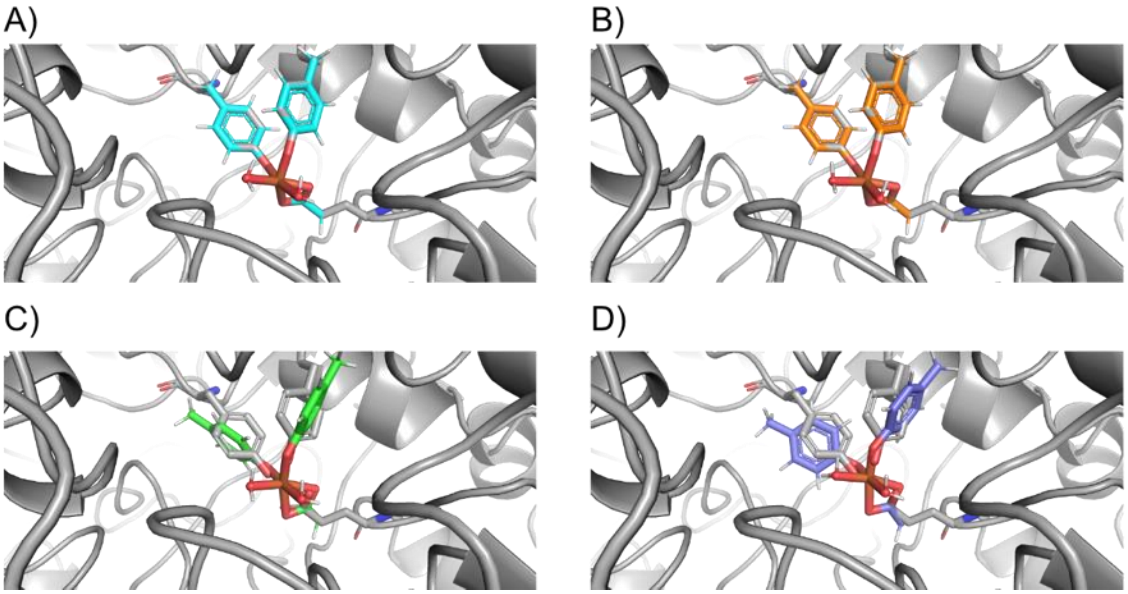

Supporting information Figure 8: The strained conformations of the binding site iron in an octahedral conformation with charges of 2 and 3 on the iron atom are given in (A) and (B), respectively. The relaxed conformations of these Fe^{2+} and Fe^{3+} complexes are given in (C) and (D), respectively.

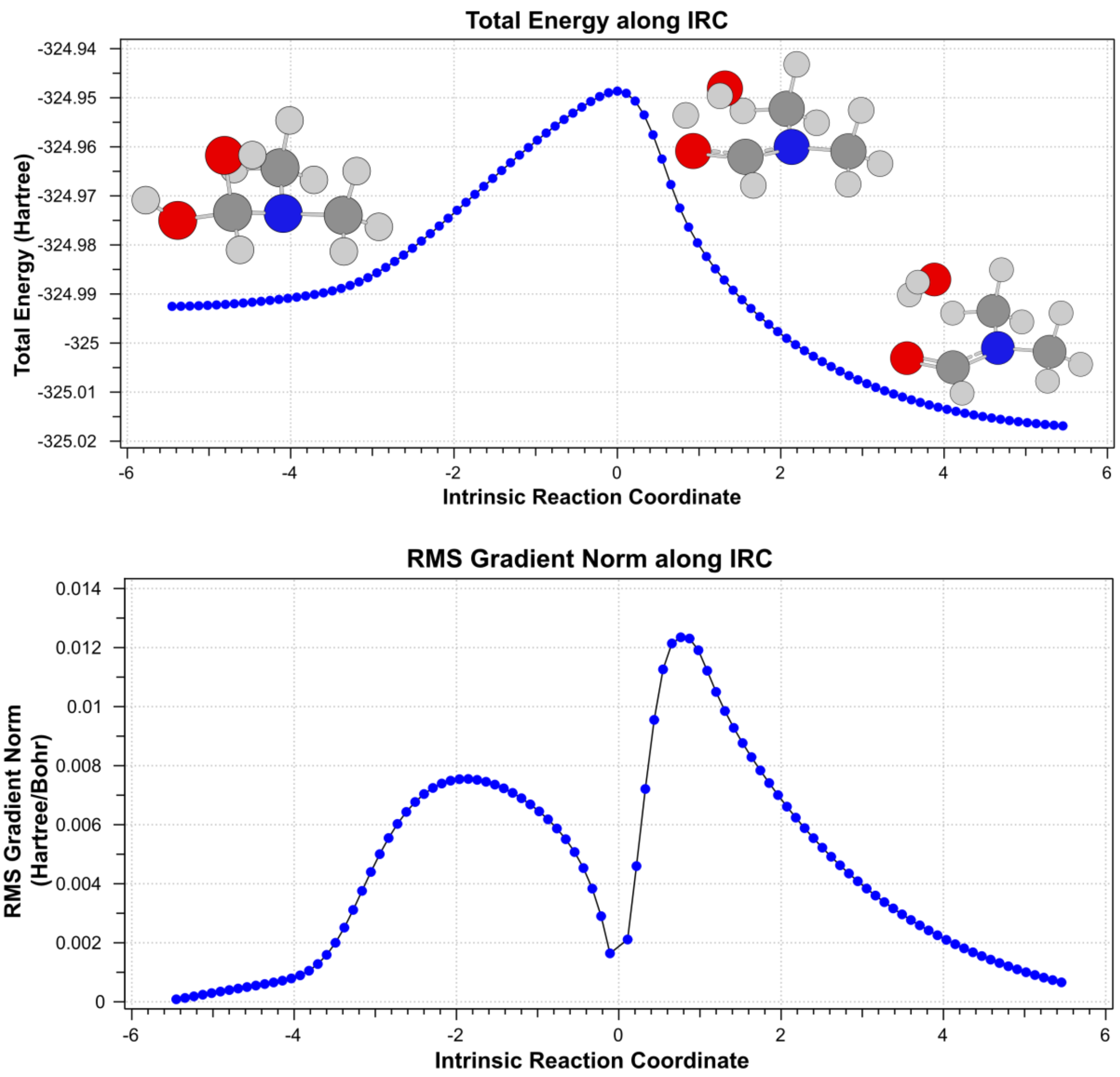

Supporting information Figure 9: The Intrinsic Reaction Coordinate (IRC) plot for the reaction between water and DMF in the gas phase as calculated at the B3LYP/6-311G(d) level of theory is given in the top half of the figure. The derivative of the IRC curve with respect to the distance coordinate is given in the bottom half. These curves and the geometries of the products and reactants confirm that the transition state found for the reaction are correct. The minimized energies for the reactants and acetal product are given in Supporting Information Table 4.
